# Validation of an Airborne Doppler Wind Lidar in Tropical Cyclones

**DOI:** 10.3390/s18124288

**Published:** 2018-12-05

**Authors:** Lisa R. Bucci, Christopher O’Handley, G. David Emmitt, Jun A. Zhang, Kelly Ryan, Robert Atlas

**Affiliations:** 1 Hurricane Research Division, Atlantic Oceanographic and Meteorological Laboratory, NOAA, 4301 Rickenbacker Causeway, Miami, FL 33149, USA; jun.zhang@noaa.gov (J.A.Z.); kelly.ryan@noaa.gov (K.R.); 2 Cooperative Institute for Marine and Atmospheric Studies, University of Miami, Miami, FL 33149, USA; 3 Simpson Weather Associates, Charlottesville, VA 22920, USA; coh@swa.com (C.O.); gde@swa.com (G.D.E.); 4 Atlantic Oceanographic and Meteorological Laboratory, NOAA, Miami, FL 33149, USA; Robert.Atlas@noaa.gov

**Keywords:** tropical cyclones, Doppler Wind Lidar, wind structure, validation

## Abstract

This study presents wind observations from an airborne Doppler Wind Lidar (ADWL) in 2016 tropical cyclones (TC). A description of ADWL measurement collection and quality control methods is introduced for the use in a TC environment. Validation against different instrumentation on-board the National Oceanographic and Atmospheric Administration’s WP-3D aircraft shows good agreement of the retrieved ADWL measured wind speed and direction. Measurements taken from instruments such as the global positioning system dropsonde, flight-level wind probe, tail Doppler radar, and Stepped Frequency Microwave Radiometer are compared to ADWL observations by creating paired datasets. These paired observations represent independent measurements of the same observation space through a variety of mapping techniques that account for differences in measurement procedure. Despite high correlation values, outliers are identified and discussed in detail. The errors between paired observations appear to be caused by differences in the ability to capture various length scales, which directly relate to certain regions in a TC regime. In validating these datasets and providing evidence that shows the mitigation of gaps in 3-dimensional wind representation, the unique wind observations collected via ADWL have significant potential to impact numerical weather prediction of TCs.

## 1. Introduction

While meteorological observations have improved in both quality and density, there remain important data-sparse regions in tropical cyclones (TCs). These regions include the boundary layer [1,2,3] and precipitation free areas, such as moats between the eyewall and outer rainbands or the eyewall and secondary eyewall [4]. One priority of the hurricane research community is to understand the physical processes and dynamics in these areas and ultimately to link them to TC genesis and intensification in order to improve TC forecasts [5,6,7].

Satellite capabilities allow remote sensing of the marine atmosphere, but the availability of remotely-sensed wind observations in and near a TC is particularly limited. Advanced Scatterometer (ASCAT) and Scatterometer Satellite-1 (SCATSAT-1), both space-based polar orbiting scatterometers, occasionally measure a snapshot of the surface wind speeds during a coincidental TC overpass. Scatterometers tend not to accurately retrieve hurricane force wind speeds because the signal used to estimate the measurement saturates over 40 m s^−1^ [8]. NASA’s newly launched Cyclone Global Navigation Satellite System (CYGNSS) also provides surface wind speed estimates [9]; however, validation of these data is still in progress. Satellite-derived atmospheric motion vectors (AMVs) provide the majority of the wind observations but are often limited to the upper troposphere [10]. Large uncertainties in the AMV height assignments lead to the utilization of fewer observations by numerical weather prediction (NWP) models. This uncertainty reduces the accuracy of the environmental vertical wind shear profile representation and can be a source of error in the forecasted intensity change of TCs [11]. Measurements between the surface and cloud tops must be observed using hurricane hunter aircraft or other non-space-based networks.

When available, hurricane reconnaissance and research aircraft provide wind measurements using various observing platforms. Although global positioning system (GPS) dropsondes are typically deployed during reconnaissance missions, they only observe discrete profiles with limited spatiotemporal coverage. The Stepped Frequency Microwave Radiometer (SFMR) provides continuous wind speed measurements but is limited to the ocean’s surface (10 m). Flight-level wind measurements are also limited to one vertical level (~3 km), similar to the SFMR observations. The tail Doppler wind radar (TDR) provides the most complete coverage of the TC wind field; however, the TDR observations are unreliable below approximately 500 m and are unable to provide measurements in precipitation-free regions [12]. The coverage of these instruments leaves several key observational gaps including the TC boundary layer.

In an effort to address observational gaps, an additional instrument, called an Airborne Doppler Wind Lidar (ADWL), can provide more frequent and accurate wind profiles in the near-storm and inner-core TC environments. The ADWL has the ability to measure line-of-sight (LOS) winds wherever its beam is pointed. It measures the Doppler shift of the backscattered return pulse along the laser beam caused by the motion of aerosols or molecules depending on the type of ADWL. There are two primary types of ADWL systems: coherent detection and direct detection [13]. Coherent detection relies on the measurement of Mie backscatter from aerosol or cloud particles. Direct detection measures the winds using both Mie and Rayleigh backscatter from aerosols and smaller atmospheric molecules. Full vector wind profiles can be retrieved given LOS measurements with multiple perspectives within the same volume. Typically, an ADWL performs a conical step-and-stare pattern looking in 8 or more different directions off of nadir. The exact horizontal and vertical resolution of the wind profiles varies depending on the power of the laser, its pulse length, and the speed at which the ADWL is travelling and scanning. This instrument has the advantage of more accurate wind measurement height assignments within an atmospheric column given the presence of aerosols, clouds, or molecules. Once the LOS is completely obscured by optically thick clouds, the signal attenuates, and accurate ADWL measurements of the wind profile are not possible in these locations—an inherent limitation of any ADWL.

The first ADWLs flown in the TC environment were in the west Pacific in 2008 as a part of The Observing system Research and Predictability Experiment (THORPEX) Pacific Asian Regional Campaign (T-PARC). A coherent ADWL was installed aboard the U.S. Naval Research Laboratory’s WP-3D Orion and circumnavigated Typhoon Nuri [14,15]. ADWL wind profiles were compared to 8 dropsondes released during the flight and showed co-located observations had good agreement with a correlation coefficient of approximately 0.98. Weissmann et al. [16] described another coherent ADWL flown 11 times on the Deutsches Zentrum für Luft- und Raumfahrt (DLR) Falcon into and around Typhoon Sinlaku over a 10-day period. A previous study [17] validated the airborne wind lidar profiles using dropsonde measurements from a field campaign sampling midlatitude synoptic-scale features in the North Atlantic. The first Atlantic hurricane coherent ADWL was flown during NASA’s Genesis and Rapid Intensification Processes (GRIP) field campaign on the DC-8 aircraft into Hurricane Earl (2010). Mechanical problems prevented a full validation of the lidar; however, some comparisons were made to a limited set of dropsondes [18]. The ADWL was repaired and successfully flown again in 2017 as a part of NASA’s Convective Processes Experiment (CPEX) into Tropical Storm Cindy [19].

The current study looks at scanning coherent 1.6-μm ADWL that was operated during the 2015 and 2016 Atlantic hurricane seasons onboard NOAA’s Lockheed WP-3D Orion (P-3). It was flown into four TCs: Danny (2015), Erika (2015), Earl (2016), and Javier (2016-East Pacific). The purpose of this study is threefold: (1) to show ADWL technology is capable of collecting frequent observations within a TC; (2) to establish an independent validation of the ADWL winds between 5 m s^−1^ and 40 m s^−1^ in a TC environment using multiple instruments; and (3) to demonstrate that the ADWL is a useful tool for observing the wind field in regions without clouds or with broken optically thick clouds.

The paper is outlined as follows. Section 2 describes the ADWL hardware and dataset. Section 3 presents a comparison of observations from instruments aboard the WP-3D and the ADWL. Section 4 discusses the complementary characteristics of the ADWL winds and finally, Section 5 presents conclusions and future work.

## 2. Materials and Methods

The P-3 ADWL has the ability to measure LOS winds above or below the aircraft. It functions similarly to a Doppler radar, but it measures the Doppler shift of the reflected photons off of aerosols in motion. The ADWL also measures the motion of hydrometeors when they are not obscured by optically thick clouds. Full wind profiles are generated based on the lidar LOS measurements and the Velocity-Azimuth Display (VAD) technique [20].

The laser and receiver are housed together in the transceiver. The P-3 ADWL operates at a wavelength of 1.6 μm with a low pulse energy of 1 mJ at a rate of 500 Hz. In the presence of aerosols, it can take measurements as close as 0.4 km and as far away as 25 km depending upon the amount of aerosol backscatter. A bi-axial scanning cylinder is mounted on the right side of the plane. It can vary its elevation angle between +120° (up and back) and −120° (down and back) and azimuthal angle between +30° (away from the plane) and −30° (underneath the plane). A fairing placed behind the scanner to reduce drag and improve streamlining prevents the barrel from pointing behind the plane. The scanner slews at 30° per second. The scanning pattern is fully programmable during flight. 

The detector signal is digitized and prepared for real-time display in a Real-time data Acquisition and Signal Processing (RASP). An analog front end (AFE) provides a first order removal of the plane’s motion based on the scanner position and information from a dedicated GPS and inertial navigation system (INS). To optimize the data collection, processing, and storage, the usual practice is to collect 5–8 kms worth of returns for a given LOS. Calibration of the instrument pointing angles was performed by flying straight and level over land before or after each mission. Given the assumption that ground returns have a wind speed of zero, the data from the flight could be further corrected. Table 1 and Zhang et al. [21] provide additional details about the instrumentation and operation mechanics.

**Figure 1:**
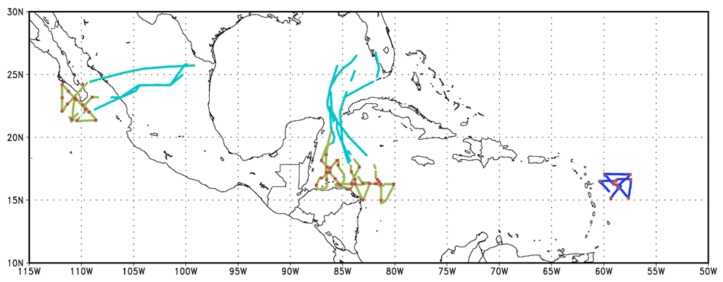
Location of airborne Doppler wind lidar (ADWL) wind profiles collected in the 2015 (dark blue) and 2016 (green and cyan) hurricane seasons. In the 2016 season, wind profiles were collected both during the transit (cyan) and in the TC environment (green). Location of dropsondes available for comparisons are also shown (red).

Figure 1 shows the location and number of profiles collected in both 2015 and 2016. A mechanical issue during the 2015 season prevented the ADWL scanner from varying its elevation angle during flight. One flight collected wind profiles into tropical storm Erika, however, it performed a different ADWL scanning pattern than the pattern used in the 5 flights completed in 2016. Therefore, the focus of this paper will be on the profiles collected during the 2016 season (Table 2). 

The ADWL performed a conical step-and-stare scan pattern around the vertical axis, 20° off of nadir. It was pointed in 12 different directions (each for 1 s) to collect horizontal winds followed by 5 s at nadir to collect the vertical wind motion. Each of the 12 directions will be referred to as a shot, or the accumulation of data collected within each 1 s dwell. The horizontal resolution of the profiles is approximately 3 km (using the estimation of 1 scan per 30 s and an aircraft velocity of about 130 m s^−1^). When the signal strength was sufficient, the ADWL was pointed upward every 2nd or 4th scan to collect winds above the aircraft.

**Figure 2:**
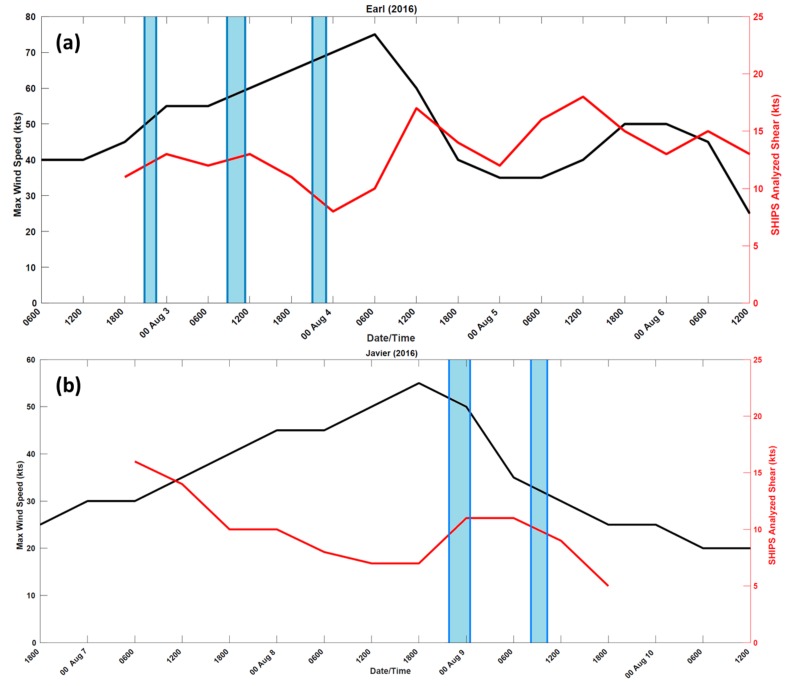
Plots of the best track intensity (black) and the Statistical Hurricane Intensity Prediction Scheme (SHIPS) diagnosed shear (red) for (**a**) Hurricane Earl and (**b**) Tropical Storm Javier. Blue highlighted regions indicate the P-3 was in the storm environment.

Data were collected in three consecutive flights occurring over 2–3 August 2016 through Earl (Figure 2a). Missions on the NOAA P-3 began that evening at 1800 UTC when Earl was a 45-knot tropical storm. Precipitation and cloud distribution were initially asymmetric because of moderate northwesterly wind shear. Gradually the shear decreased which allowed for a more symmetric distribution of convection and intensification to a category 1 hurricane.

Two additional flights (Figure 2b) collected data in Tropical Storm Javier on 8–9 August 2016 in the East Pacific off the coast of Baja California. During the first mission, the mid-level vertical shear increased causing the mid- and low-level circulations to decouple and weaken the storm. Javier continued to rapidly weaken to a 35-knot tropical storm as it encountered both dry air and mountainous terrain in the Baja Peninsula by the second mission.

## 3. Results

The WP-3D aircraft uses several instruments that collect horizontal wind measurements in a TC environment. Wind profiles the ADWL collected in 2016 are compared to GPS dropsondes, SFMR, flight-level wind probe, and the TDR. It must be noted that the WP-3D coherent detection ADWL coupled with precise navigation and attitude information provides an inherently accurate LOS measurement (<0.5 m s^−1^ root mean squared error). Most of the differences in vector profiles between wind observing systems can be attributed to sampling differences, especially near tropical cyclones.

### 3.1. Importance of Number of Line-Of-Sight Shots in Airborne Doppler Wind Lidar Data

**Figure 3:**
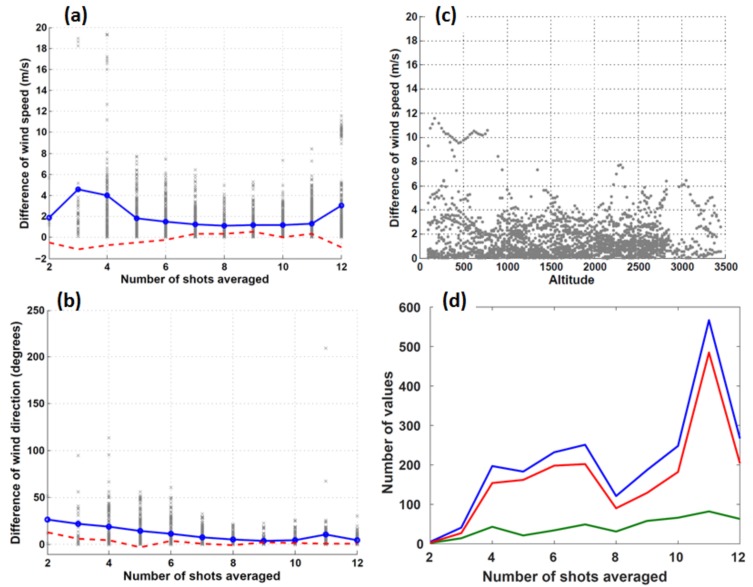
(**a**) The absolute difference between collocated lidar and dropsonde wind speeds (gray x symbols) as a function of number of line-of-sight (LOS) shots collected to determine the measurement. The standard deviation (solid blue) and bias (dashed red) of these differences are shown; (**b**) Same as (**a**), but with ADWL and dropsonde wind directions outside of the tropical cyclone (TC) center; (**c**) The absolute difference between collocated lidar and dropsonde wind speeds with 5 or more LOS shots as a function of altitude (m); (**d**) Line graph of the number of observation comparisons for the wind speeds (blue), wind directions outside the TC center (red), and wind directions in the TC center (green) as a function of number of LOS shots collected to determine the measurement.

Observations from the ADWL are determined by the representation of the wind in a volume in space defined by the LOS measurements obtained. Figure 3a,b shows the difference between the dropsonde wind speeds (direction) and nearby ADWL measurement as a function of the number of LOS observations. The largest differences between wind speed measurements, up to 20 m s^−1^, occur when the ADWL has fewer shots to retrieve a full wind vector. The majority of the ADWL observations obtained are calculated using 5 or more LOS shots (Figure 3d). The set of outliers calculated using 12 LOSs (Figure 3a) will be discussed in Section 3.2. Removing data calculated using less than 5 LOS shots reduces the standard deviation by more than 50 percent, which suggests wind speeds utilizing 5 shots or more tend to be more reliable. This result is consistent among all validating instrument data including GPS dropsondes, flight-level winds, Stepped Frequency Microwave Radiometer, and Tail Doppler Radar. For this reason, the discussion of all subsequent results refers to ADWL observations using 5 or more LOS shots.

### 3.2. Global Positioning System Dropsonde Data

Dropsondes provide a profile of wind speeds and directions below the aircraft with a vertical resolution of about 5 m as determined by GPS location [22]. Table 2 shows the number dropsondes deployed in each storm. A total of 2056 paired samples of ADWL and dropsonde winds are obtained from the 5 flights in 2016. These pairs are selected based on criteria that collocates observations in both space and time. The average separation of paired observations is 2.064 km and limited to a maximum separation of 5 km from each other. As a result, paired dropsondes and ADWL profiles are temporally separated by no more than 6 minutes.

**Figure 4:**
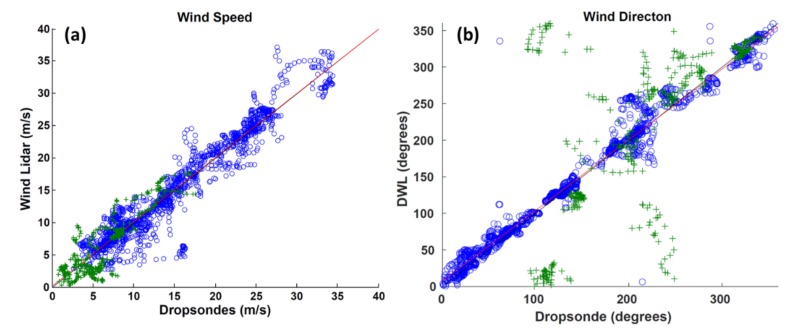
(**a**) Comparison of collocated ADWL and dropsonde wind speed observations (m s^−1^) outside of the TC center (blue o symbols) and within the TC center (green + symbols). Also shown are the line of best fit (red) and the line of perfect correlation (black); (**b**) Same as (**a**) but with collocated ADWL and dropsonde wind directions. ADWL winds are calculated from 5 or more LOS shots.

Figure 4a features a correlation scatter plot of paired ADWL and dropsonde wind speed measurements. The least squares best fit of the ADWL data is:

S_ADWL_ = 1.0026(S_DROP_) − 0.0202, (1)

where S_ADWL_ is the wind speed (m s^−1^) measured by the ADWL and S_DROP_ is the wind speed (m s^−1^) measured by the dropsonde. The correlation coefficient was 0.9586. Focusing on the set of outliers mentioned in Section 3.1 (Figure 3a) reveals that these pairs are associated with one dropsonde and ADWL profile comparison. Though the dropsonde and ADWL profile are separated by an average 4.55 km, the average wind speed difference is 10.27 m s^−1^. Figure 3c shows these outliers also occur within 1 km of the surface. One explanation for this large discrepancy could be the difference between the nature of the measurements. The ADWL represents volume-averaged observations while the dropsonde is an advected point measurement. The dropsonde from the outlying comparison was released just prior (approximately 2 minutes) to fixing the center of Tropical Storm Earl. This area closer to the radius of maximum wind is typically where the wind speeds in a TC can change drastically over relatively small distances. The volume-averaged observation from the ADWL could have smoothed a high-gradient region while the point measurement from the dropsonde sampled the instantaneous wind field.

Figure 4b shows the comparison of wind directions for the same set of 2302 paired samples in 2016. The bias was 1.868, degrees, RMSE was 40.66 degrees, and correlation coefficient was 0.917 for all the data. The dataset was then divided into two regions: one at the TC center and one outside the TC center. Figure 4b highlights why a comparison between the ADWL and dropsonde in a high gradient region should be made cautiously. The majority of the outliers are associated with comparisons made close to the storm center (i.e., eye); an area of high directional gradient. The comparison at regions close to the storm center does not appear to be improved by the number of shots used to retrieve the ADWL wind direction (not shown). 

Outside of the TC center region, the comparison between ADWL and dropsonde wind direction show good agreement in the remaining 1652 paired observations (Figure 4b). The least squares best fit to the data is

D_ADWL_ = 0.9659(D_DROP_) + 7.5271. (2)

The bias was 2.42, degrees, RMSE was 16.626 degrees, and correlation coefficient was 0.9858. Figure 3d shows that 90% of the wind direction measurement comparisons are made outside the TC center and with a sufficient number of LOS shots.

### 3.3. P-3 Flight Level Data

**Figure 5:**
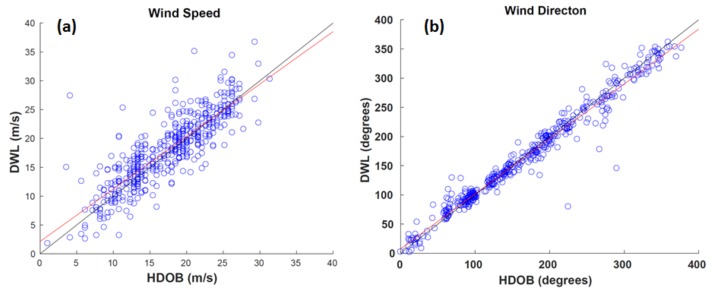
Comparison of collocated ADWL and high-density observation (HDOB) wind observations. (**a**) Wind speed (m s^−1^) comparison shown with the line of best fit (red) and the line of perfect correlation (black); (**b**) Same as (**a**), but with wind directions (degrees).

The WP-3D is equipped with flight-level data sensors that collect a variety of atmospheric measurements at high temporal frequency (up to 40 Hz), including the wind speed and direction. These measurements are averaged at 30-s intervals to create high-density observations (HDOBs) and are regularly transmitted during flights [23]. Observations (HDOBs) are compared to the nearest ADWL measurement within 5 km horizontally and 500 m vertically (Figure 5a). This results in 469 paired observations with an average horizontal and vertical separation of 1.04 km and 388 m, respectively. The least squares best fit to the wind speed data is

S_ADWL_ =0.9093(S_HDOB_) − 2.1437. (3)

The bias was 0.553 m s^−1^, RMSE was 3.3119 m s^−1^, and correlation coefficient was 0.8461. There is an improvement in the statistics when 6 instead of 5 LOS shots are used to estimate the ADWL wind speed. The bias, RMSE, and correlation coefficient improve to 0.499 m s^−1^, 2.811 m s^−1^, and 0.8884, respectively. This suggests that while limiting the number of LOS shots to 5 removes many outliers it is not a universal threshold and will likely need to be revisited when the ADWL dataset is more substantial.

Wind directions are compared using the same set of observations (Figure 5b). The least squares best fit to the wind direction data is

D_ADWL_ = 0.9411(D_HDOB_) + 7.5749. (4)

The bias was −2.2147 degrees, RMSE was 16.63 degrees, and correlation coefficient was 0.9828. The agreement between ADWL and HDOBs may be related to the effective smoothing of these data. The ADWL completes a scanning pattern in approximately the same amount of time the HDOBs are averaged. The relatively long integration time smooths out features that are inherently difficult for the ADWL to capture, like the wind direction shift in the TC center. Unlike comparisons to dropsondes, no correlation exists between the measurement difference and the location of the observation relative to the TC center or the radius of maximum wind. There is also no correlation to the horizontal or vertical separation of the measurements.

### 3.4. Stepped Frequency Microwave Radiometer Data

**Figure 6:**
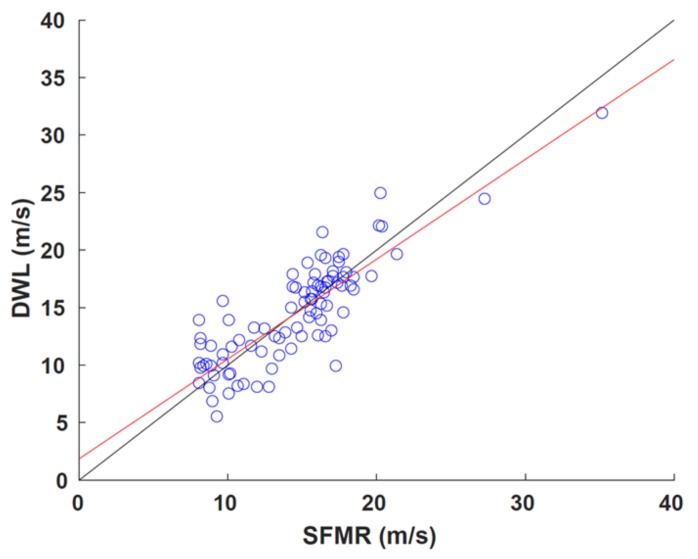
Comparison of collocated ADWL and stepped frequency microwave radiometer (SFMR) wind speed observations (m s^−1^). Also shown are the line of best fit (red) and the line of perfect correlation (black).

The SFMR measures the nadir brightness temperatures, T_B_, at six C-band frequencies along the flight track. A retrieval algorithm relates T_B_ to ocean surface wind speed to produce surface wind speed estimates of approximately 1–2 km horizontal along-track resolution [24]. The RMSE associated with SFMR wind speeds is 3.9 m s^−1^ using the algorithm from Klotz and Uhlhorn [25]. The ADWL measurements closest to the surface are compared to SFMR surface wind speeds (Figure 6). The ADWL measurement needed be at least 300 m or lower to be included in the comparison. SFMR wind speeds below 8 m s^−1^ were removed because they are considered to be less reliable [26]. This created a sample size of only 90 paired measurements.

The ADWL winds were adjusted by a factor of 0.83 to account for reduction in wind speed due to friction within the boundary layer [21]. The average vertical separation was 110.5 m. The least squares best fit to the data is

S_ADWL_ = 0.8676(S_SFMR_) + 1.8604, (5)

with a correlation coefficient of 0.847. There was no correlation between wind speed difference and rain rate or horizontal or vertical separation of the measurements. The small sample size limits the interpretation of this comparison. A more robust interpretation can be determined when more data are collected.

### 3.5. Tail Doppler Radar Data

**Figure 7:**
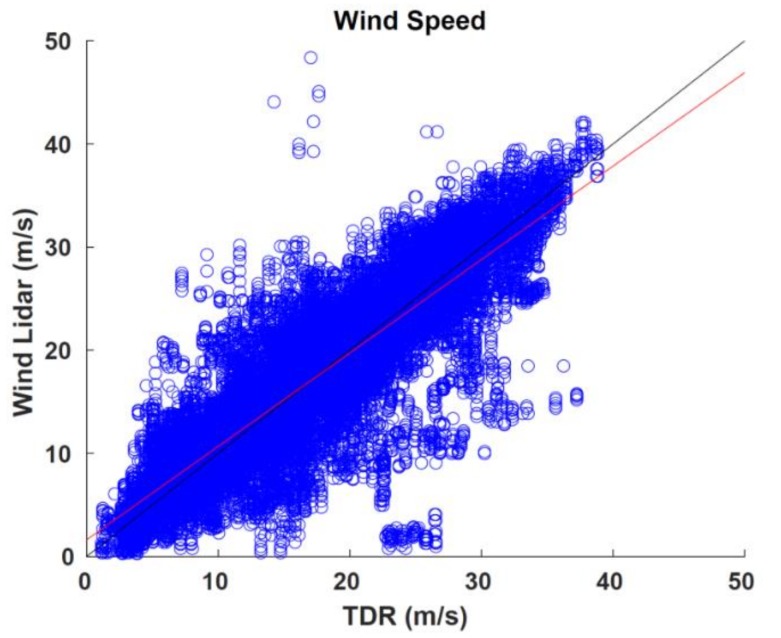
Comparison of collocated ADWL and tail Doppler wind radar (TDR) wind speed observations (m s^−1^). Also shown are the line of best fit (red) and the line of perfect correlation (black).

The TDR is an X-band (3.2 cm wavelength) radar which scans conically in the vertical to the fore and the aft of the aircraft. An automated variational synthesis of the Doppler radial velocities [27] creates wind profile analyses with horizontal and vertical resolutions of 1.5 km and 150 m, respectively, along the flight track while the aircraft performs a cross section of the TC. These analyses are compared to the coinciding ADWL wind profiles regridded to the TDR horizontal resolution (Figure 7).

There are 40,559 paired observation comparisons. The bias was −0.0434, RMSE was 4.1335, and correlation coefficient was 0.8604. The least squares best fit to the data is

S_ADWL_ = 0.8061(S_TDR_) + 1.6118. (6)

Given the large sample size, there are many more outliers even with the restriction of 5 or more LOS shots to produce an ADWL vector wind. 85.4% of the comparisons are within 5 m s^−1^ of each other, 11.6% are between 5 to 10 m s^−1^, and 3% are greater than 10 m s^−1^.

The majority of the largest differences are associated with a larger average and maximum aircraft roll measurements during the ADWL pattern sequence. In differences of 10 m s^−1^, the maximum roll was 9.28 degrees compared to 4 and 4.44 degrees for differences of 5–10 m s^−1^ and less than 5 m s^−1^, respectively. This indicates that the plane was likely experiencing more turbulence and not maintaining a straight and level attitude. Deviations from the straight and level could be a possible explanation of these large outliers. Another potential cause for an increase in number of outliers may be related to the opposing nature of the regimes best suited for each instrument. The optimal ADWL signal is retrieved in clear-air or optically thin, broken clouds as opposed to the TDR’s retrieval in rain and optically thick clouds.

### 3.6. Comparison of Validation Results

The validation results of 2016 ADWL data is displayed in Table 3, comparing error statistics analyzed using each instrument. Winds from the HDOB flight-level probe resulted in the best correlation, where the effective smoothing of length scales appears to be the primary reason for this result. Although dropsondes tend to be the standard by which validation of aircraft data are performed, the difference in measurement procedure limits the ability for multi-scale wind speeds to be validated. The outliers discussed in Section 3.2 reflect the inconsistency of measurement scale by comparing observations collected in high gradient regions. Nonetheless, the correlation between dropsondes and ADWL profiles was 0.9586 and 0.917 (0.9858) for wind speed and direction (direction outside TC core), respectively. A similar thought process may exist for SFMR validation, but the small sample size prevents a rigorous comparison. Finally, the largest collection of paired observations, those validated using TDR data, showed the lowest correlation among instruments which may be due to a number of factors discussed in Section 3.5. Overall, the correlation coefficients of ADWL range between 0.84 to 0.98 and reflect a positive validation of these data.

## 4. Discussion

One of the primary goals of the WP-3D ADWL was to reduce the observation gap present in TC observing systems. The asymmetric cloud distribution in both TCs in 2016 gave the ADWL the opportunity to collect observations in precipitation-sparse regions that were largely unsampled by the other airborne instrumentation. This section explores cases of how the ADWL filled in new information about the TC wind field given our confidence in the ADWL measurements based on the results in the previous section.

**Figure 8:**
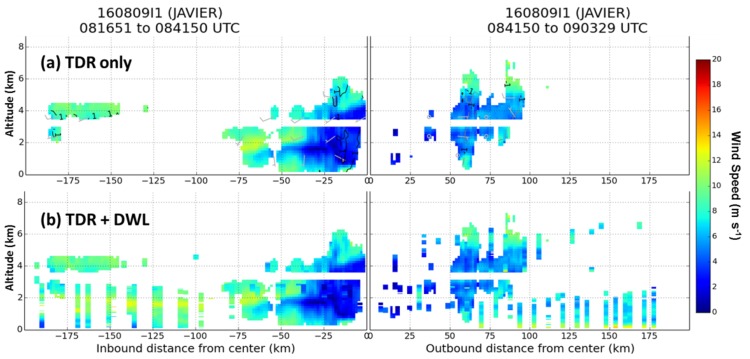
Comparison of a cross section through the center of Tropical Storm Javier on 0840 UTC 9 August 2016 with wind speeds (m s^−1^, shaded) collected by (**a**) the TDR only and (**b**) the TDR and the ADWL.

An example of improved coverage was in Tropical Storm Javier as it rapidly weakened off the coast of Baja California at 0900 UTC 9 August 2015. Figure 8 shows a comparison of the wind coverage in a cross section of the storm with TDR winds only versus ADWL and TDR wind profiles. The addition of ADWL observations significantly increases the coverage of winds. A previously unobserved wind maxima between 100 and 175 km from the center on the inbound leg at 1000–2000 m altitude is detected by the ADWL. The ADWL provides an additional 2900 new wind observations in this particular cross section. The addition of these data allows for a more complete representation of the wind field affecting TCs and their evolution.

**Figure 9:**
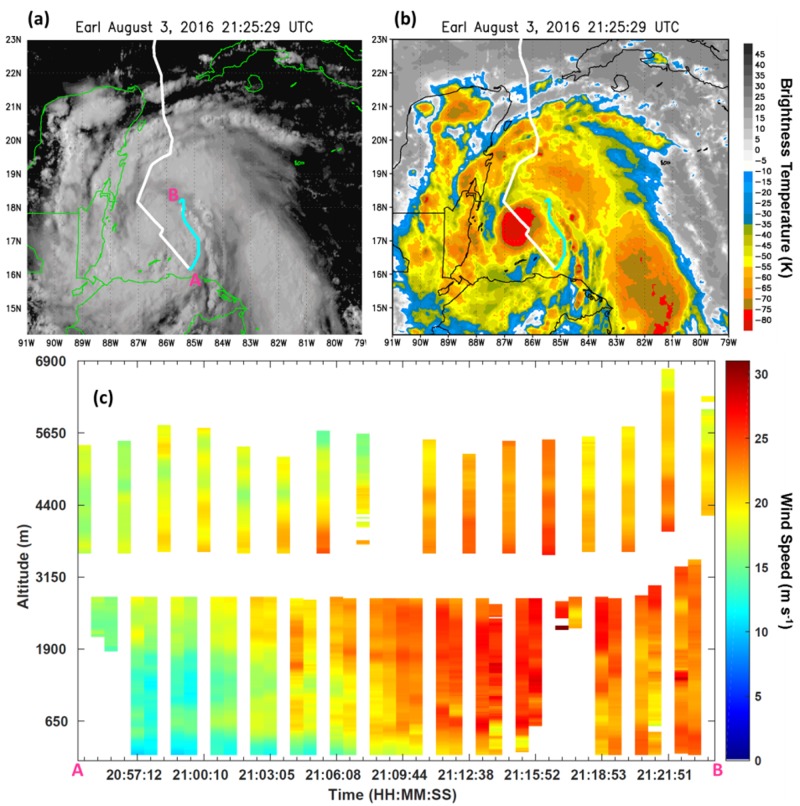
(**a**) Visible and (**b**) IR satellite imagery of Hurricane Earl at 2125 UTC 3 August 2016 with the P-3 flight track overlaid (white) and the downwind leg (cyan). Star denotes the TC center. (**c**) ADWL wind speed profiles collected during the downwind leg of the flight.

The research aircraft can modify its sampling strategies to enhance coverage of complementary observing systems. An example of this occurred in Hurricane Earl on 1800 UTC 3 August when the aircraft flew parallel to a developing rainband. The ADWL was able to capture wind profiles in the moat region outside of the convection (Figure 9) while the TDR collected observations within the expanding convection of the rainband. Flight patterns such as these maximize the observing capabilities of both systems. Future studies could repeat this sampling strategy to better understand the convective evolution of rainbands. When operating the ADWL in a more mature, well-developed hurricane, it would be beneficial to consider circumnavigating the center in a moat region between convective rainbands to increase the collection of wind profiles. Observations in this largely unobserved region may be important for internal processes involving eyewall replacement cycles.

**Figure 10:**
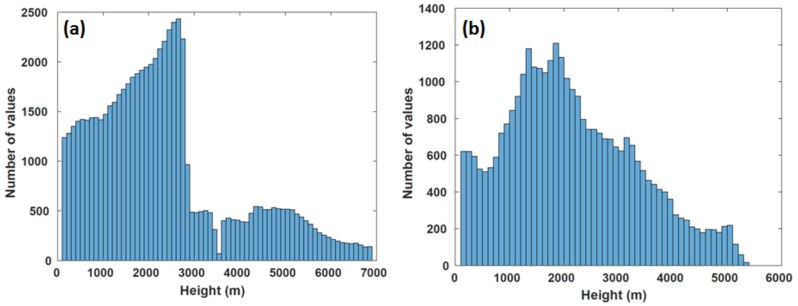
Histogram of ADWL measurements during the 2016 season (**a**) in the TC inner core environment and (**b**) during the transit to and from the TC.

In addition to the collection of TC inner core wind profiles, the ADWL also collected almost 900 wind profiles during the transit to and from the storms. These profiles provided wind information between the vertical levels of 150 and 5500 m (Figure 10) with the most observations within the upper portions of the boundary layer (1500–2500 m). In addition to enhancing the physical understanding of environmental conditions known to impact TCs, the collection of these wind measurements could also be used in numerical models to provide supplemental information about the near-storm environment. Observing System Simulation Experiment (OSSE) studies [28] reflect this interpretation, which concluded that assimilating synthetic lidar data has the potential to improve TC track and intensity forecasts.

## 5. Conclusions

The WP-3D Airborne Doppler Wind Lidar proved to be a reliable wind observing system in the tropical cyclone environment. It continuously collected measurements in all five of the missions flown in the 2016 Atlantic hurricane season. Altogether, 1630 wind profiles were collected in the inner core, near-storm region, and during transit. These data span from 100 m above the ocean surface up to 7 km with the majority collected at the top of the boundary layer between 1500 to 2500 m.

The ADWL wind profiles compared well to other onboard wind observing instruments, namely dropsondes, flight-level wind probes, SFMR, and TDR. Collocated comparisons between ADWL and dropsonde winds had correlation values of 0.9586 and 0.917 for wind speed and direction, respectively. The correlation values of DWL profile wind measurements among all instruments remain relatively high, thus validating the data for future use to complement the existing airborne dataset. Most outliers in the ADWL wind profiler dataset were objectively identified based on the number of LOS observations used to estimate the wind vector where at least 5 shots are preferred. Remaining outliers emphasize the need for additional spatial and temporal information.

Wind profiles from the ADWL should be used carefully in high-gradient regions. By design, the ADWL averages over a volume and smooths wind speed and direction within the observed volume. This was shown to be problematic when observing the wind direction shift in regions close to the TC center. It may also under-represent the peak wind speeds in high-gradient regions, which is an inherent limitation in performing the volumetric profiler procedure.

The ADWL can provide both complementary and new observations in the TC environment by collecting data in regions largely unobserved by other instrumentation. When operated in conjunction with the TDR, these instruments can collect wind observations in both precipitation and clear-air. This creates a more comprehensive, symmetric coverage of the TC wind field and its surrounding environment. Increased 3-dimensional coverage gives the ADWL substantial potential to improve hurricane prediction by providing numerical models a more complete representation of the atmospheric winds.

Future studies aim to mitigate some of the limitations presented here and investigate the potential for ADWL observations to impact hurricane prediction. For example, the individual LOS observations used to create these wind profiles contain valuable information within that volume, allowing for small-scale phenomenon to be sampled. Additional studies will compare the use of wind profiles to the LOS measurements within a numerical model framework with the goal of optimizing the use of these data. Finally, as the number of ADWL cases increases, a more rigorous investigation can aid in improving the understanding of TC track, intensity, and structural evolution.

## Figures and Tables

**Table 1:** Main attributes of the lidar system.

Wavelength	1.6 µm
Energy per pulse	1.5 mJ
Pulse length (full width half maximum)	80 m
Repetition rate	500 Hz
Telescope diameter	0.10 m
Scanner (cylindrical)	±120° elevation; ±30° azimuth
Nadir angle	20°
Vertical resolution (20% overlap)	50 m
Horizontal resolution	~3–6 km
Time per revolution	30 s
Number of shots per scan	12
Accumulation time per stare	1 s
Accumulated pulses per stare	500

**Table 2:** Overview of the 2016 Airborne Doppler Wind Lidar measurements.

Storm Name	Date	Storm Strength (m s^−1^ (kts))	Time in Storm (h)	ADWL Profiles in Storm		Transit Time (h)	ADWL Profiles in Transit	Dropsondes
				Up	Down			
Earl	2 August 2016	23.2 (45)	1.82	39	158	5.58	159	8
Earl	3 August 2016	28.3 (55)	2.57	54	223	4.83	207	10
Earl	3 August 2016	33.4 (65)	2.75	99	246	4.55	137	14
Javier	8 August 2016	25.7 (50)	3.13	70	233	4.6	125	8
Javier	9 August 2016	18 (35)	2	37	161	4.6	271	9
**Total**			**12.27**	**299**	**432**	**24.16**	**899**	**49**

**Table 3:** Summary of the 2016 Airborne Doppler Wind Lidar validation statistics.

Dataset	Number of Paired Observations	Observation Type (Units)	Bias	RMSE	Correlation Coefficient
All dropsondes	2056	Speed (m s^−1^)	0.0155	2.373	0.9586
	2056	Direction (°)	1.8680	40.6569	0.917
Dropsondes without centers	1652	Direction (°)	2.42	16.626	0.9858
HDOBs	469	Speed (m s^−1^)	0.553	3.3119	0.8461
	469	Direction (°)	−2.2147	16.63	0.9828
SFMR	90	Speed (m s^−1^)	−0.0606	2.4577	0.847
TDR	40559	Speed (m s^−1^)	−0.0434	4.1335	0.8604
